# Biomedical Scientists' Perceptions of Ethical and Social Implications: Is There a Role for Research Ethics Consultation?

**DOI:** 10.1371/journal.pone.0004659

**Published:** 2009-03-02

**Authors:** Jennifer B. McCormick, Angie M. Boyce, Mildred K. Cho

**Affiliations:** 1 Stanford Center for Biomedical Ethics, Stanford University, Palo Alto, California, United States of America; 2 Department of Pediatrics, Stanford University, Palo Alto, California, United States of America; 3 Department of Medicine, Mayo Clinic and College of Medicine, Rochester, Minnesota, United States of America; 4 Department of Science and Technology Studies, Cornell University, Ithaca, New York, United States of America; Aga Khan University, Pakistan

## Abstract

**Background:**

Research ethics consultation programs are being established with a goal of addressing the ethical, societal, and policy considerations associated with biomedical research. A number of these programs are modelled after clinical ethics consultation services that began to be institutionalized in the 1980s. Our objective was to determine biomedical science researchers' perceived need for and utility of research ethics consultation, through examination of their perceptions of whether they and their institutions faced ethical, social or policy issues (outside those mandated by regulation) and examination of willingness to seek advice in addressing these issues. We conducted telephone interviews and focus groups in 2006 with researchers from Stanford University and a mailed survey in December 2006 to 7 research universities in the U.S.

**Findings:**

A total of 16 researchers were interviewed (75% response rate), 29 participated in focus groups, and 856 responded to the survey (50% response rate). Approximately half of researchers surveyed (51%) reported that they would find a research ethics consultation service at their institution moderately, very or extremely useful, while over a third (36%) reported that such a service would be useful to them personally. Respondents conducting human subjects research were more likely to find such a service very to extremely useful to them personally than respondents not conducting human subjects research (20% vs 10%; chi^2^ p<0.001).

**Conclusion:**

Our findings indicate that biomedical researchers do encounter and anticipate encountering ethical and societal questions and concerns and a substantial proportion, especially clinical researchers, would likely use a consultation service if they were aware of it. These findings provide data to inform the development of such consultation programs in general.

## Introduction

Progress in the biomedical sciences may have significant societal impact through potential benefits, but also potential risks. The questions being asked by biomedical scientists encompass topics of social sensitivity [1], such as the tension between stem cell research and religious groups, or debates about a genetic basis of race. In addition, new ethical issues are raised about the conduct of research, such as what kinds of research findings, if any, should be returned to individual research participants and how to involve communities in study design. Scientists are increasingly being asked to consider the broader impacts of their research, motivated by an higher societal demand for the accountability of and justification for scientific research.[2] Some, including other researchers, are calling upon members of the scientific community to take a more proactive role in addressing the ethical and societal implications of their research.[3]–[5] But do they have the necessary tools, resources and willingness to do so?

To facilitate the involvement of biomedical scientists in addressing ethical, societal, and policy considerations related to their research, the Stanford Center for Biomedical Ethics launched the Benchside Ethics Consultation Service (BECS) in 2005.[6] This program, as well as similar services started by other institutions [7] has been loosely modelled on clinical ethics consultation, as described in detail in other publications [8], [9] (although it is not limited to clinical researchers). As such, it is intended to serve an advisory, rather than regulatory or oversight function, and is client-driven. Programs like this are likely to multiply as dozens of research institutions join the U.S. National Institutes of Health Clinical and Translational Science Award program, which has a research ethics component. As part of Stanford's BECS program, we conducted a preliminary study to determine what kind of need scientists perceive for such a service.

Although there is an increasing literature on scientists' views of scientific misconduct and behavior,[10]–[12] very few studies have been published on scientists' perceptions of the ethical, societal, and policy ***implications*** of their research.[13], [14] To the best of our knowledge, there is no published study on biomedical researchers' attitudes toward the implications of their research or toward research ethics consultation. To better understand how researchers in the biomedical sciences perceive ethical, societal, and policy issues, how they should be addressed, and by whom, we conducted a large multi-phase study comprised of interviews, focus groups, and surveys. We examined how societal and ethical concerns or questions related to their research were perceived, whether researchers would be open to consulting a bioethicist about such a concerns, and how useful biomedical science researchers would find a research ethics consultation service.

## Results

### Respondent Characteristics

#### Telephone interviews

We completed semi-structured interviews with 16 of the 20 individuals we contacted between May–June 2006. Three were graduate students, 2 postdoctoral fellows, 5 research staff, 4 instructors, and 2 faculty and they represented departments of genetics, biological sciences, pathology, biophysics, biochemistry, psychiatry, pediatrics, cancer biology, applied physics, neurology, and obstetrics/gynecology.

#### Focus groups

Our five focus groups were conducted over the course of three weeks between August and September 2006. Our initial focus group was conducted as a pilot and consisted of four individuals who responded to a list serve request for volunteers. Twenty-five of the 120 individuals we contacted for the 2 hour focus groups agreed to participate in the subsequent four focus groups: 7 postdoctoral/clinical fellows, 7 graduate students, 4 senior research staff, 2 instructors, and 5 faculty. These individuals represented an array of departments and programs including but not limited to biological sciences, microbiology, plant biology, molecular pharmacology, radiology, pathology, biochemistry, proteomics, psychiatry, genetics, medicine, and hematology.

#### National Survey

Of the 2000 individuals to whom we sent surveys, we made contact with 1707 individuals, and of these achieved a 50% response rate. Nearly three-fourths of our survey respondents came from institutions with medical schools and almost one half of our respondents were from universities a with bioethics presence (see Table 1). In addition to the five departments we sampled, our respondents self-reported affiliations with a range of other departments, such as medicine, microbiology, animal science, biophysics, bioengineering, and physiology.

**Table 1:** Survey respondent population.

Characteristics	Percent of survey respondents
	N (%)
***Position***	
Faculty	282 (34%)
Research staff	132 (16%)
Postdoctoral fellow	183 (22%)
Graduate student	223 (27%)
***Type of research***	
Uses human subjects	280 (33%)
Uses vertebrate animals	330 (42%)
Uses hESC	21 (3%)
Basic research	685 (83%)
Clinical research	164 (20%)
Translational research	164 (20%)
Applied research	212 (26%)
***Institutional characteristics***	
With medical school	583 (71%)
With bioethics presence	376 (46%)

Based on self-report, most of our survey respondents were faculty, followed by graduate students, postdoctoral/clinical fellows, and research staff, but these differences in response rate were not statistically significant (see Table 1). One third (33%) of our survey respondents reported conducting research involving human subjects (including all researchers using human subjects, de-identified human data, or both and hereafter we refer to this category simply as ‘human subjects’). Over 40% reported using vertebrate animals, while only 3% indicated they were conducting human embryonic stem cell research (see Table 1). When asked to report the type of research they do, over 80% included basic research, about 20% included clinical research, another 20% included translational research, and just over 25% included applied research in their response. Responses were not mutually exclusive and we did not define these terms.

### Is there awareness of, and need for, research ethics consultation?

One premise of a client-driven ethics consultation service is that researchers themselves identify ethical and societal questions related to their work, however defined. To explore this awareness issue, in our survey we queried researchers on how often they have encountered or anticipate encountering certain ethical and societal questions, without explicitly defining “ethical and societal questions”. Forty-one percent of our survey respondents reported that they have never had ethical or societal questions arise as a result of their research. However, over one third (36%) of respondents said that they have had such questions arise 1–2 times while 23% reported such concerns arising 3 or more times in the course of their career. Fifty-three percent of respondents do not anticipate that the research they are currently conducting or planning to conduct will generate a question or concern about a societal or ethical issue related to their research. Almost one third (31%) of our survey respondents agreed that the research they are currently conducting or planning to conduct *might* generate such a question or concern while 17% *definitely* anticipate that their current or future research will generate questions or concerns about societal or ethical issues. We also looked at responses to these two questions by position. Faculty more often reported having encountered such concerns more than once than those in other positions (37% faculty, 17% research staff, 22% postdoctoral fellows, 23% graduate students, Pearson chi^2^ p = 0.005). More faculty also reported *definitely* anticipating their research to generate questions or concerns than the other positions (45% faculty, 19% research staff, 13% postdocs, 23% graduate students, Pearson chi^2^ p = 0.004).

### Would biomedical science researchers consult bioethicists about ethical and societal issues?

An important indicator of researchers' willingness to use a research ethics consultation service is whether researchers look to bioethicists as a source of expertise and would be willing to talk with them about concerns. Although only 8% of survey respondents reported having talked about ethical and societal implications or issues with a bioethicist, over 25% of survey respondents reported that they would talk to a bioethicist *about a specific concern*. Our discussions with researchers support this finding. Very few interview and focus group participants explicitly said they have talked to bioethicists about these issues in general, although those that had were positive about their experiences. One faculty expressed in an interview an appreciation of specific bioethics colleagues' “open door policy… [they] allow me to pick their brain when necessary, and I've never felt that I had nowhere to go when I had a sensitive question.”

Tests for dependence between the variable ‘position’ and the question about willingness to talk to a bioethicist about a concern showed that there is a significant association (Pearson chi^2^ p<0.001). Indeed, our survey data also show that 47% of all ‘yes’ responses to the question were from faculty while 13% were research staff, 18% were postdoctoral fellows, and 22% were graduate students. This finding corroborates some of the statements made to us by our interviewees. For example, one postdoc we interviewed noted “…to be frank and honest, … the thing that would keep me from going … would be more the possible consequences … if I went for a consultation there, I could get into trouble with my collaborators, I could get into trouble with my boss and that kind of stuff….” suggesting that junior researchers are less comfortable speaking to authority figures who are not their principal investigators.

In addition, our survey data show that researchers who used human subjects were more likely to say that they would be comfortable talking to a bioethicist about an ethical or societal concern (32%), compared to 22% of those not using human subjects in their research (Pearson's chi^2^ p = 0.004).

### How useful would biomedical science researchers find a research ethics consultation service?

Based on our focus groups and interviews with researchers, we found that they are generally open to the principle of a research ethics consultation service and saw utility in the service. There was a tendency for researchers to see the service as more useful to others, rather than themselves personally. As one professor we interviewed stated “Well, I think it's useful for the university, absolutely. Certainly the types of projects that I'm doing now wouldn't require that type of service. But, you know, if things came up, then certainly.” Our survey asked how useful researchers at the respondent's institution would find a research ethics consultation service, and how useful the respondent *personally* would find such a service.

Approximately half of all the survey respondents thought that a research ethics consultation service would be moderately to extremely useful to researchers at their institutions while 49% said it would not be useful or would be only slightly useful (see Table 2). A somewhat smaller percentage, just over a third, reported that the service would be moderately to extremely useful to themselves personally (see Table 2). Overall, survey respondents saw a research ethics consultation service more useful to one's institution (mean = 2.57 on Likert scale of 1–5 where 1 = not at all and 5 = extremely) than to themselves personally (mean = 2.33 on a Likert scale of 1–5) (Wilcoxon sign rank, p<0.001).

**Table 2:** Usefulness of a research ethics consultation service.

Question	Categories	Totals, N (%)	Not at all, N (%)	Slightly, N (%)	Moderately, N (%)	Very, N (%)	Extremely, N (%)
How useful would researchers at your institution find a research ethics consultation service?							
	Total	814 (100%)	68 (8%)	330 (41%)	311 (38%)	93 (11%)	12 (2%)
	Human subjects researchers	269 (100%)	14 (5%)	93 (35%)	111 (41%)	43 (16%)	8 (3%)
	Nonhuman subjects researchers	544 (100%)	54 (10%)	237 (44%)	199 (37%)	50 (9%)	4 (1%)
How useful would you personally find a research ethics consultation service?							
	Total	831 (100%)	178 (21%)	333 (40%)	203 (24%)	97 (12%)	20 (2%)
	Human subjects researchers	273 (100%)	35 (13%)	97 (36%)	85 (31%)	42 (15%)	14 (5%)
	Nonhuman subjects researchers	557 (100%)	143 (26%)	236 (42%)	118 (42%)	54 (10%)	6 (1%)

More survey respondents using human subjects stated the service would be moderately to extremely useful to them personally (51%) than did respondents not using human subjects (32%) (Pearson chi^2^ p<0.001). Furthermore, of respondents using human subjects, 20% said the service would very to extremely useful to them personally compared to 10% of respondents not using human subjects. The mean response for all survey respondents using human subjects was 2.64+/−0.064 on a Likert scale of 1–5 while the mean response for all respondents not using human subjects was 2.18+/−0.041 (Kruskal-Wallis, p<0.001). While the means between these two types of researchers varied across institutions, the trend was similar – within a given institution more researchers using human subjects reported thinking a research ethics consultation service would be more useful to them personally than did researchers not using human subjects.

In addition to our survey data, qualitative data from our conversations with researchers suggest that many make an immediate association between using human subjects and ethical and societal implications. As one clinical instructor we interviewed said, “in the process of doing my research, there are always ethical issues that come up… Any time you work with people, you come in contact with issues that you wonder, am I doing the right thing?” A faculty we interviewed stated, “If people are doing cutting edge genetic research and mixing human and animal cells, or stem cells, there may be issues people want discuss, but the type of research that I'm doing, I don't think is at the cutting edge of ethical issues. But I think if I were to move into doing clinical studies, then I think those ethical issues are much more significant.”

Of all survey respondents, those from psychiatry departments were more likely to find a research ethics consultation service moderately to extremely useful personally than those from other departments (61% vs 36%; Pearson chi^2^ p<0.001). We did not find this to be true for any other department in our sample. However, we did find that those from biological sciences departments were more likely to find a research ethics consultation service ***not at all*** useful or ***slightly*** useful personally (68% vs 58%; Pearson chi^2^ p = 0.01). We are also particularly interested in understanding whether a medical school presence or a bioethics presence has any influence on how biomedical researchers perceive of ethical and societal implications related to their research, bioethicists, and research ethics consultation. Our sampling does not allow us to make definitive statements based on our current data about institutional factors such as the presence of a medical school or bioethics center or program. We did, however, find that in general, there exist differences among the seven institutions in our sample. For example, Pearson chi^2^ analyses show slight differences by institution in responses to the question asking how often questions about ethical and societal implications of research come up (never/not at all: 32% to 49%, 1–2 times: 28%–43%, >5 times: 9%–16%, p = 0.012) and anticipation of encountering an ethical or societal questions of concern (yes: 11%–30%, maybe: 27%–40%, p = 0.002).

## Discussion

### Is there a need for research ethics consultation?

A surprisingly large fraction of survey respondents reported encountering an ethical or societal question that arose from their own research. Thus, there is a potentially large user-base for a research ethics consultation service.

The finding that more faculty reported having encountered or anticipating ethical or societal questions was not surprising given that faculty generally have had longer careers and more research experience than others in our sample. We hypothesize that researchers from institutions with a medical school presence or a bioethics presence will be more attuned to ethical and societal implications and thus more likely to anticipate such questions or concerns arising from their research.

Based on our qualitative data from interviews and focus groups we had expected the fraction of postdoctoral/clinical fellows reporting to anticipate encountering questions or concerns in current or planned research to be higher. Postdoctoral/clinical fellow participants in focus groups and interviews appeared to be very engaged in identifying potential ethical and societal implications as well as interested in discussing them. Data from our small pilot survey (n = 64) show that postdoctoral fellows as a group thought a research ethics consultation service would be more useful and they would be more likely to use such a service than faculty, research staff, and graduate students in that sample (McCormick, J.B., Boyce, A.M., and Cho, M.K., unpublished data).

Finally, when we introduced the phrase “ethical and societal implications” we attempted to place no value on the word “implications”. That is, we wanted to determine what researchers think implications are, and whether they think of either positive or negative or both kinds of implications. Because we did use “implications”, “questions” and “concerns” interchangeably, there might have been some tendency for study participants to think of only negative implications. However, we used the phrase “ethical and societal implications” based on findings from a pilot survey sent to 150 genetic researchers. In that survey we had used “ethics” – research ethics, ethical implications, etc. We found that while some pilot survey respondents seemed to think broadly about research ethics, many seemed to consider only issues related to the conduct and misconduct of research, such as plagiarization, authorship, and animal welfare. By including the word “societal” we hoped to indicate to researchers – without being leading, that we were open to hearing about broader ethical and societal issues and concerns, as well as issues in the responsible conduct of research.

Our data indicate that participants in our study did not necessarily universally link “negative” with “social implications”. We found that some researchers were neutral, for example, thinking of developing drugs for third world diseases or curing cancer or increasing food production. Admittedly, there was likely a leaning toward thinking of negative social implications by many study participants, but it was not our intention to imply that.

### Would biomedical science researchers consult bioethicists about ethical and societal issues?

Our data suggest that faculty were more likely to report that they would consult a bioethicist about a societal or ethical issue. This is not surprising given that faculty are more likely than postdoctoral/clinical fellows and graduate students to have formal opportunities to interact with colleagues outside of their discipline, e.g. faculty committees, cross-disciplinary teaching assignments, etc. and built larger social networks within the academic community. The survey data however do not allow us to draw conclusions about differences between senior faculty and junior faculty. There is often a perceived hierarchy in science; graduate students, postdoctoral fellows, and junior research staff may sometimes feel compelled to “check-in” with their principal investigator (PI) before seeking consultation or advice from someone not in the lab.[15], [16] Junior investigators might feel apprehension about suggesting an ethical or societal concern exists because these kinds of discussions do not occur routinely and are not always encouraged in the lab. In addition, junior faculty may be hesitant to point out an ethical or societal concern to a more senior faculty member, especially if tenure has not been obtained or if that senior faculty holds a departmental or institutional administrative position. This suggests that research ethics consultants be sensitive to the hierarchical and power dynamics that can exist in a research environment. The potential for such personal dynamics make it likely that consultants will need to engage in greater outreach senior level researchers and faculty to encourage open discussion with junior researchers, as well as with junior faculty and trainees, in order to make them feel more comfortable seeking advice from bioethicists. It will be crucial for such outreach efforts to be keenly cognizant of junior/senior level relationships, as well as for additional work to be done to identify what might be effective in reaching junior level researchers.

Our data also show an association between using human subjects in research and an openness to talking to bioethicists about concerns. Researchers who use human subjects in their research acknowledge that they themselves think about issues such as privacy, safety, and incidental findings, and even researchers not using human subjects were quick to acknowledge the inherent societal and ethical implications of using humans in research. This might be in part due to the training human subjects researchers must undergo on a regular basis. However, it is also possible that some researchers who interact with human subjects, especially in the context of clinical trials, are able to more easily perceive the direct impact their research has on others.

Researchers who interact with the humans who participate in their studies (e.g. consenting participants, collecting the samples from participants, administering investigational drugs to participants, interviewing participants, etc) might have a deeper appreciation of how the work they do (or don't do) can impact an individual life. This notion was mentioned briefly during one of our focus groups as one postdoc mentioned this in the context of the IRB: “Yeah, the protection of human subjects, IRB is very relevant to my work. I'm not an MD, but I have to consent patients for my study every week, and that reminds me of what the impact of my work is supposed to be on society. It can be encouraging and discouraging at the same time, and it reminds me of why I'm interested in cancer research and why I want to develop better treatments for cancer.”

### How useful would biomedical science researchers find a research ethics consultation service?

Our survey data support the qualitative data we collected previously: researchers are apt to see the usefulness of a research ethics consultation service generally, but might have more difficulty identifying how such a service might be of use to them personally. This difference could be due to how respondents defined ethical and societal concerns and what kinds of issues they perceived a consultation service to address. For example, those who perceived a consultation service to resolve issues of scientific misconduct might not have felt that such a service would apply to themselves. A question that remains is what kinds of institutional factors might influence researcher attitudes toward a research ethics consultation service, e.g. medical school presence, bioethics presence.

Nearly half of our survey respondents said a research ethics consultation service would not be useful at all, or only slightly useful, to researchers at their institution. This might be expected given that research ethics consultation services are a new phenomenon and many of our participants were learning about them for the first time. Because of their unfamiliarity with the concept of a formal ethics consultation service for researchers, it is possible that building trust between scientists and consultants will be important to the success of the services. We would suggest that 51% of our respondents recognizing that a research ethics consultation service would be useful is positive. Researchers can sometimes conflate compliance and regulation with ethics and considering ethical and societal implications. Data from our study suggest that some the researchers have trouble recognizing social and ethical issues, and that some feel the scientists themselves can handle social and ethical concerns that arise, along the lines that the scientific community can regulate itself. We explore such barriers to using a research ethics consultation service and even thinking about ethical, societal, and policy implications in general elsewhere.

Indeed, from our qualitative data, we have found that there is a wide range of views on what ‘ethical and societal’ concerns, questions, or implications mean to researchers. For some, they refer to scientific misconduct, authorship, and conflict of interest issues. For others, it means access to health care, protecting the environment, and resource conservation. In between, we found that some researchers have concerns about reporting research findings on specific populations without stigmatizing the population, how to handle supposedly de-identified human data, and what to do with a research subject's “abnormal” genetic information that arises during the course of a research study for which there is no known resolution. We are currently systematically analyzing this spectrum of “definitions”, and our preliminary findings suggest that, in addition to a broad view of what constitutes ‘ethical and societal concerns or questions’, research that is seen as ‘publically or politically controversial’ is identified as having ethical and societal implications. We recognize that this wide variation in views of ‘ethical and societal’ concerns, questions, or implications might have influenced some of our survey responses. For instance, over half of our survey respondents indicated that an ethical or societal question related to their research had arisen at least once in the course of their career. This large fraction might be due to the very broad range of perspectives researchers have of what an ‘ethical and societal’ question is. On the other hand, more respondents might have indicated that they had encountered such concerns, questions, or implications if they had had a broader view of ‘ethical and societal’.

### Limitations

Here we have been able to examine the relationship between individuals' characteristics and their responses toward the questions we asked. Our preliminary finding of institutional differences suggest that in addition to individual characteristics, there might be some aspects of the nature of the individual institutions at play, e.g. a medical school presence, a bioethics presence, and we are currently examining how these variables might be associated with individuals' response.

We relied on publicly available websites, which are often not kept up to date, to create our sample population from which our sample was derived. This might contribute to our 15% non-contact rate and help explain why our survey respondents self-identified as being affiliated with departments other than those we initially targeted.

Finally, there is a tendency to associate the phrase ethical and social implications (or questions or issues) with something negative. This indeed was not our intent when we presented the questions to our study participants; rather we wanted ethical and social implications to viewed neutrally.

### Conclusions

Our findings demonstrate that biomedical researchers recognize ethical and societal questions and concerns during the course of their work, suggesting that there may be a need for research ethics consultation services, and that there is a small but significant subset of researchers that might use such a service. We also found that there is openness to talking to a bioethicist about such concerns, especially among faculty, and that there is a general acceptance of the idea of a research ethics consultation service at an institutional level.

Research ethics consultation services might need to engage in greater outreach among junior faculty, postdoctoral fellows and students in order to increase their comfort level in consulting a bioethicist – someone they might perceive as being an outsider to the research community, unfamiliar on a personal level, or intimidating if seen as too senior. In addition, additional outreach might be necessary to engage researchers who do not use human subjects.

This type of service is likely to become much more widespread in the U.S. through the implementation of the National Institutes of Health Clinical and Translational Science Awards, primarily aimed at clinical researchers. However, as these services become institutionalized, and as funding agencies such as the National Science Foundation, require applicants to discuss the ethical impact of their proposed research, they could become utilized by basic scientists as well. Such services might be used internationally as the EU Seventh Framework Programme, similar to NSF, requires its applicants to discuss the ethical and societal implications of the proposed research, and the NIH Fogarty International Center has launched an initiative aimed at supporting the development and expansion of curricula in international bioethics about conducting international research as well as training individuals to serve in the capacity of a bioethics reviewer of research protocols in low- and middle-income countries. The increased interest in funding agencies in ensuring ethical and societal considerations are given in the development and process of research clearly indicates that additional empirical work is warranted to determine what cultural and economic differences might exist and how to best harmonize the variation.

## Methods

We conducted telephone interviews and focus groups with researchers from Stanford University and a mailed survey of researchers at 7 different universities in the United States. All studies, including the procedure for obtaining informed consent, were approved by the Stanford institutional review board. Interview participants provided consent by agreeing to schedule and participate in a phone interview. The letter inviting potential interviewees to participate explained the goals and risks and benefits of the study as well as the process for obtaining consent. In addition, all interviewees were informed of the goals and risks and benefits of their participation and asked to provide verbal consent at the start of the interview. Focus group participants were provided a copy of the informed consent in the letter inviting them to participate in the study. They were given a second copy and the opportunity to ask questions about the study, and asked to sign the form at the start of the focus group. Individuals responding to the national survey provided consent by returning a completed or partially completed survey. The letter inviting them to participate, which accompanied the survey, explained the goals and risks and benefits of the study as well as the process for obtaining consent.

### Telephone interviews

We invited 20 researchers who came from a range of departments and programs chosen to represent individuals conducting basic and clinical sciences research. These included genetics, biological sciences, cancer biology, psychiatry, pathology, biochemistry, and biophysics from Stanford University to participate in a brief phone interview. These researchers were selected using stratified random sampling from a database created from publicly available websites of Stanford University life science departments. We stratified by position, selecting four individuals from each of five categories: graduate students, postdoctoral fellows, research staff, clinical instructors, and faculty. Interviews lasted 15 minutes to 45 minutes. All interviewees provided consent for recording and transcribing the interviews and received a $10 book store gift card with the letter inviting them to participate in the study.

We were particularly interested in learning whether the interviewees ever think about ethical and societal implications related to the biomedical sciences and what these thoughts might be. Specific questions in the interviewer guide were: Tell me about the research you do; What are some of the ethical, social, and policy implications related to your research that you think about; To whom do you talk about these kinds of issues; and, To whom might you go for advice if you had an ethical or societal concern or question related to your research. We also wanted to learn whether our study participants were aware of the Stanford Benchside Ethics Consultation Service (BECS) and if so, what they thought about it. If they were not aware of BECS, we then described it as a research ethics consultation service that had recently been established at Stanford before asking what they thought about it. In addition, the letter inviting researchers to participate in our interview study included a brief description of a research ethics consultation service. The interviews were semi-structured and conducted by three different trained individuals over the course of approximately three weeks. The questions were piloted on two volunteers, a faculty member and a graduate student.

### Focus groups

We conducted a pilot focus group comprised of 2 research staff and 2 postdoctoral fellows contacted and recruited through a Stanford University-wide list serve. The focus group lasted one hour and the volunteers received breakfast and a $5 gift card. Four 2 hour focus groups were conducted and were comprised of 5–7 individuals each. These researchers were selected by stratified random sampling from the same database developed for the telephone interviews. One of these focus groups consisted of graduate students, one of postdoctoral/clinical fellows, and two of a mix of faculty, clinical instructors, and senior research staff. Approximately 120 individuals were initially invited to participate in one of the four 2 hour focus groups, and those who did participate received lunch and a $75 gift card. All participants provided consent to have the focus groups audio-recorded and transcribed.

We wanted to gain a better understanding of what comes to mind for researchers when they hear the phrase “ethical and societal implications related to their research” or “ethical and societal implications of the life sciences in general”. We specifically did not provide participants with a definition of “ethical” or “societal” as one of our aim was to determine how broadly, or narrowly, researchers think when hearing the phrase “ethical and societal implications related to their research”. Specifically we posed these questions: People have a lot of different views on and definitions of “societal and ethical issues” related to biomedical science research… what are the societal and ethical issues related to biomedical science research that keep you awake at night? What about things that come up in your day-to-day research? How often do you have to deal with some of the issues like the ones we've been discussing? To whom do you talk about these issues? Are there enough forums for researchers to discuss these types of issues? We explored the focus group participants' thoughts on the role of bioethics and controversy in research by prompting with the following questions: What's your take on the role of bioethicists? How about your take on the role for scientists in dealing with societal and ethical issues? What do you think makes research controversial? What kinds of research are controversial? How does controversy influence research? What is your opinion on the role of scientists in dealing with controversy? We also presented a specific ethical and societal issue in biomedical research in the form of a scenario and asked for participants' thoughts and reactions to it. One example we used was researchers' obligations to report results of potential medical relevance to individual research participants, in particular when there is some uncertainty associated with the finding.

### National survey

Seven research universities were selected for the national survey, including Stanford University and six others, chosen as follows: Using a publicly available list from the National Institutes of Health (NIH), we identified the top 100 U.S. university NIH awardees in 2004. Two of these universities were eliminated – one because English is not the main language and the other because it is actually a National Laboratory. The remaining 98 were placed into one of 6 categories based on the following institutional attributes: public vs. private institution, medical school presence vs. no medical school presence, and bioethics presence vs. no bioethics presence. A university was considered to have a “bioethics presence” if, using the search engine on the university's homepage and the search terms ‘bioethics department’, ‘bioethics center’, OR ‘bioethics program’, there was an identifiable group of individuals of 2 or more whose research or teaching interests include bioethical issues. To be of significance, this identifiable group had to appear in the search (conducted in April 2006) within the first 20 hits. One university was randomly selected from each of the six categories.

Two thousand paper surveys, four pages long and consisting primarily of closed-ended questions were mailed to faculty, research staff, instructors, postdoctoral and clinical fellows, and graduate students. We limited our sample to biomedical science researchers from five departments at each institution: biochemistry, biological sciences, and genetics (non clinical) and pathology and psychiatry/behavioral sciences (clinical). Genetics and psychiatry/behavioral sciences were selected because they are areas where ethical and societal issues are often raised. The remaining departments were selected as follows: Stanford University life science departments were categorized as either clinical or non clinical, randomly sorted, and two selected from non clinical and one from clinical. Closely corresponding departments at each of the selected universities were identified and publicly available websites were used to create a database for each institution selected. As before, we stratified by position before random sampling equally from each position category. Because one goal of the study was to ascertain the perceptions of Stanford University biomedical scientists to bioethics, bioethicists, and a research ethics consultation service, we oversampled Stanford researchers, mailing 500 surveys to this group, while 250 were mailed to researchers at each of the remaining six institutions. A $5 gift card to a local coffee shop was enclosed with the national survey and a letter inviting them to participate in the study.

The survey consisted of four sections that focused on: issues around the relationships between life science research and society; science communication and public engagement; politics and policy-making in the sciences; and background information. The data from our interviews and focus groups were used to help formulate many of the survey questions. Most of the questions were aimed at identifying perceptions and attitudes toward bioethics and bioethicists, including whether respondents would talk to bioethicists, what kinds of societal and ethical questions they might have or anticipate encountering, and how useful respondents thought a research ethics consultation service might be. As with the interviews, we prefaced the questions about a research ethics consultation service with a brief description what that might be. In addition the letter that accompanied the survey included a brief description of a research ethics consultation service. The question formats included multiple choice, Likert scales, ordered category items, and ranking of choices from a list created based on data from our earlier work.

### Analysis

Our survey results were analyzed using descriptive statistics, Pearson's chi^2^, Wilcoxon sign rank, and Kruskal-Wallis mean rank tests in STATA. For analysis of our qualitative data from interviews, focus groups, and the survey, we used a grounded theory approach,[17] implemented with MaxQDA qualitative software. To analyze the qualitative data, three individuals identified themes and recurring and emerging topics and the relationships between them and developed a set of codes and definitions. Data were independently coded by at least two coders and final coding derived by consensus [18].
